# Feeding dairy cows for improved metabolism and health

**DOI:** 10.1093/af/vfac059

**Published:** 2022-10-14

**Authors:** Leoni F Martins, Derek E Wasson, Alexander N Hristov

**Affiliations:** Department of Animal Sciences, The Pennsylvania State University, University Park, PA, USA; Department of Animal Sciences, The Pennsylvania State University, University Park, PA, USA; Department of Animal Sciences, The Pennsylvania State University, University Park, PA, USA

**Keywords:** acidogenic diet, amino acids, energy, inflammation, leaky gut, phytonutrients

**Implications**

- Maximizing intake is key for maintaining gut health.
- Feeding highly acidogenic diets in the prepartum might be detrimental to energy balance in the postpartum period.
- Phytonutrients (e.g., phenolic compounds) may improve intestinal and overall cow health.
- Future research should aim to integrate energy, protein, and mineral metabolism in transition cows.

## Introduction

**Figure 1.**
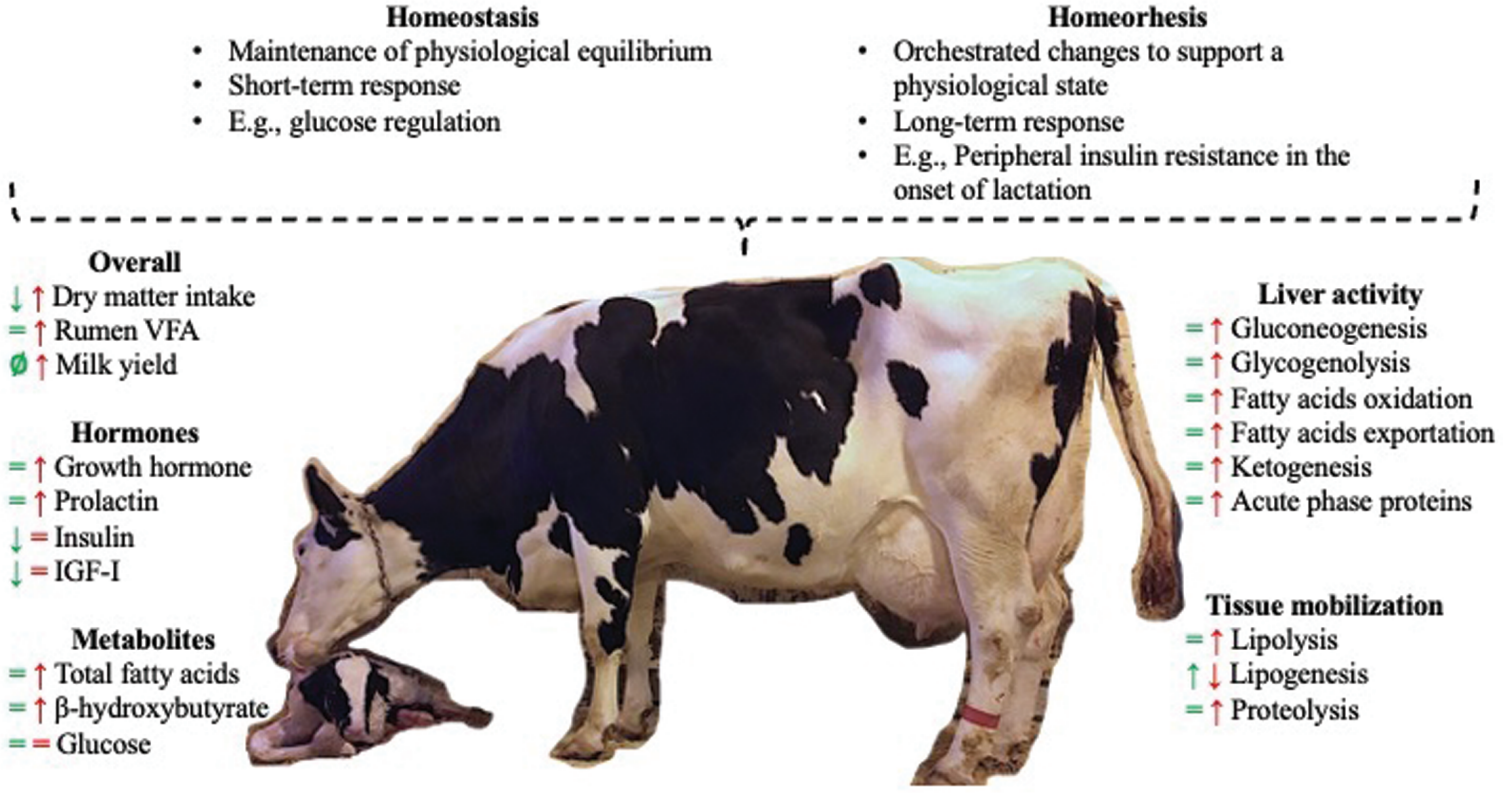
Diagram summarizing the main effects of homeostatic and homeorhetic controls occurring in healthy dairy cows during pre-(green symbols on the left side) and postpartum (red symbols on the right side). Adapted from Bauman and Currie (1980). VFA = volatile fatty acids.

Increasing production efficiency of dairy cows through improved health is not possible without adequate nutrition. Hippocrates (460–370 BC), the father of modern medicine, apparently claimed that all diseases begin in the gut, and this might be appropriately applied to dairy cows. The transition period, defined as 3 wk before and 3 wk after parturition, is characterized by extensive metabolic and physiologic changes mediated by homeostatic and homeorhetic processes (Figure 1; Bauman and Currie, 1980), and the use of nutritional and management strategies during this period can provide long-lasting effects for dairy cows. For instance, failing to manage the calcium status of the animal during the transition period can be detrimental to milk production in the subsequent lactation. Multiparous cows with delayed or persistent hypocalcemia measured at days 1 and 4 postpartum produced less milk (up to 7.2 kg/d in the first 6 wk of lactation) than cows with transient or no hypocalcemia (Seely et al., 2021).

Early-lactation milk production is positively associated with milk production in the entire lactation, whereas for each kg of milk yield increase in peak lactation, total milk production (i.e., 305-d) was increased by 157 kg in healthy cows (*R*2 = 0.69; Mellado et al., 2011). Conversely, total milk production was decreased by 410 kg when dairy cows were diagnosed with a clinical disease (Carvalho et al., 2019) in the first 21 d in milk (DIM).

Approximately 25% of cows leave the herd in the first 60 DIM (Fetrow et al., 2006) and the culling risk in the first 120 DIM is 12% greater for cows diagnosed with any metabolic disease compared with healthy cows (Probo et al., 2018). In addition to hypocalcemia, ketosis and fatty liver are the main syndromes occurring in modern dairy cattle and feeding strategies can be used to minimize the prevalence of these metabolic diseases while maximizing the performance of dairy cows (Ingvartsen, 2006; Ingvartsen and Moyes, 2013). Emerging theories on the etiology of these metabolic diseases may be tightly linked to dry matter intake (DMI) and immune dysfunction, which can be also improved by the manipulation of nutrients in diets.

The objectives of this review are to 1) provide an overview of how inflammation is related to the metabolism and nutrition of dairy cows; 2) present some nutritional approaches that can be applied to modulate the immunometabolism of dairy cows; 3) point-out the state-of-the-art and the frontiers in knowledge regarding how these nutritional approaches (e.g., acidogenic diets, controlled energy diets in the prepartum period, amino acid supplementation in the prepartum period, and eugenol, cinnamaldehyde, capsaicin, hemp, and macroalgae supplementations) interact with metabolism and immune system in dairy cows. The role of other nutritional approaches on the immunometabolism of dairy cows (e.g., essential fatty acids, methyl donors, yeast, and other phytonutrients supplementation; Lopreiato et al., 2020), and other feeding and management strategies (e.g., dietary manipulation in the postpartum period, body condition score, and use of propylene glycol; Ingvartsen, 2006; Ingvartsen and Moyes, 2013) have been extensively discussed and it is beyond the scope of this review.

## Inflammation, Metabolism, and Nutrition of Dairy Cows: an Integrated System

The majority of the cellular components of a human’s immune system are located within the gastrointestinal (GI) tract (West et al., 2014). Assuming cows are similar, the GI tract serves as the primary site of interaction between the animal and the outside world. The intestinal barrier is responsible for absorption of nutrients from the digesta and the deterrence of pathogenic microorganisms and their endotoxins. Absorption of endotoxins (e.g., lipopolysaccharide; LPS) across the intestinal lumen can lead to immune activation and inflammation. The inflammation cascade is characterized by increases in body temperature, circulating neutrophils and lymphocytes, endothelial blood flow, and cytokine expression. Expression of cytokines, tumor necrosis factor-α, and interleukin (IL)-1β, have also been identified as likely inducers of hypophagia exhibited during disease onset (Brown and Bradford, 2021). Intestinal barrier integrity is essential in deterring these antagonists and can be compromised by the endotoxins themselves, and/or limited DMI. Dysfunction of the barrier and the subsequent immune activation is known as a leaky gut syndrome and is believed by some as an alternative explanation for the etiology of ketosis and hypocalcemia. Kvidera et al. (2017b) determined that feed restriction (60% of feed intake) increased circulating endotoxin and acute phase proteins, along with intestinal histology indicative of intestinal barrier dysfunction in lactating Holstein cows. Additionally, Kvidera et al. (2017a) observed similar onset of leaky gut syndrome in pair-fed cows compared with cows with suppressed intakes caused by intestinal barrier damage from gamma-secretase inhibitor administration. In the first case, the compromised barrier integrity of the pair-fed cows was solely caused by feed restriction.

**Figure 2.**
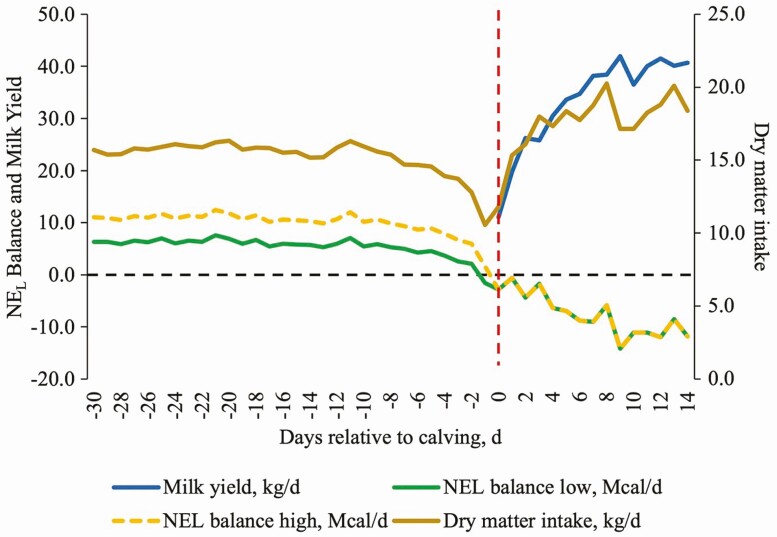
Simulation of average dry matter intake (DMI), milk yield (MY), and balance of net energy for lactation (NE_L_) in dairy cows (*n* = 45) fed low (1.35 Mcal/kg) or high (1.65 Mcal/kg) energy diets prepartum, and 1.70 Mcal/kg of NE_L_ postpartum. Considering that DMI and MY were not affected by dietary energy concentration prepartum, cows fed a higher energy diet had a higher decrease of NE_L_ at calving (day 0) compared with cows fed a lower energy diet. In a practical scenario, dairy cows will experience a negative energy balance in the first weeks of lactation because the rate of increase in milk production is greater than the rate of increase in DMI.

The negative effects of decreased DMI are particularly problematic when considering the stereotypical intake depression observed in transition cows (Figure 2), which are already immunocompromised during the onset of calving (Sordillo, 2016). Additionally, at the time of transition, peripartum diets commonly have increased levels of energy in the form of starch, which can lead to sub-acute ruminal acidosis (SARA). Indeed, feeding additional starch (Haisan et al., 2020; Shi et al., 2020) or concentrate prepartum (Penner et al. 2007) neither improves metabolic adaptation nor mitigates the risk of ruminal acidosis postpartum. Passage of rumen undegraded starch and endotoxins produced by the onset of SARA continues downstream, causing further damage, increased hindgut fermentation of carbohydrates, and transportation of endotoxin across the intestinal barrier (Gressley et al., 2011). In turn, absorption of endotoxin prompts an inflammatory response and decreases intake in a feedback loop detrimental to the cow’s health status.

## Immune Activation Requires Energy and Calcium Availability

Inflammation and immune activation come at a metabolic cost to the animal. Activated immune cells undergo a metabolic switch in mammals, becoming obligate glucose utilizers and with increased insulin sensitivity to facilitate rapid uptake of glucose, while peripheral systems become insulin resistant (Calder et al., 2007). The amount of energy required during an immune response remains difficult to quantify due to a multitude of intrinsic variables (e.g., severity and duration of immune response, environmental conditions, basal metabolic levels, and stage of lactation). A study conducted with pair-fed cows, immune activated with LPS or not (i.e., control), sought to quantify the amount of glucose required and to differentiate between endotoxin-induced and restricted DMI-induced immune activation (Kvidera et al., 2017b). Consequently, researchers conservatively estimated that during the first 12 h of an endotoxin response the immune system of a lactating Holstein cow consumes > 1 kg of glucose, and that redirection of glucose to immune cells comes at the expense of other energy demanding metabolic processes (e.g., milk production). In addition to increased glucose requirements, calcium metabolism is also affected by immune activation. Horst et al. (2020) demonstrated that 13.7 g of Ca were necessary to maintain eucalcemia for the first 12 h following an LPS challenge in dairy cows. Additionally, in a retrospective study classifying a cohort of lactating Jersey cows without clinical diseases, prolonged, low-grade inflammation cows (i.e., cows with continuously elevated blood haptoglobin concentration measured on days 4 and 7 postpartum) represented 25% of the animals evaluated, and they had reduced blood calcium concentration and milk production (i.e., –2.3 kg/cow/d) across the first 14 and 60 DIM, respectively (Martins et al., 2021). Thus, even clinically healthy dairy cows might experience a certain level of subclinical inflammation.

## Feeding Strategies to Improve Immunometabolism

### Acidogenic diets in the prepartum period diet

Acidogenic diets (i.e., negative dietary cation-anion difference; DCAD) are known to cause a compensated, mild metabolic acidosis in animals. The DCAD is calculated based on the content of selected positive and negative dietary mineral ions (e.g., Na^+^, K^+^, Cl^−^, and S^−^) expressed in mEq/kg (Ender et al., 1971). Feeding negative DCAD diets is an effective way to decrease the incidence of clinical hypocalcemia (i.e., milk fever; Lean et al., 2019). Although the incidence of milk fever is lower than 6% in the United States, subclinical hypocalcemia (that is considered a gateway disease to the development of other health issues) is still detected in 25% and 50% of primi- and multiparous dairy cows, respectively (Reinhardt et al. 2011). Reducing DCAD from +200 to –100 mEq/kg in multiparous cows increased blood ionized calcium (iCa) concentration in a meta-analysis by Santos et al. (2019). Ionized calcium plays a role in neutrophil activation in response to inflammatory stimuli. Therefore, feeding acidogenic diets might enhance innate immune function, although the mechanisms are not completely elucidated, and results are controversial. Phagocytosis capacity and oxidative burst of neutrophils were improved in healthy and sick cows fed a negative DCAD diet (–130 vs. +130 mEq/kg; Martinez et al., 2018), whereas neutrophil function did not differ in healthy cows supplemented with diets ranging from –112 to –100 mEq/kg, compared with control in commercial herds (Serrenho et al., 2020).

Responses to supplementation of acidogenic diets during the prepartum period might interact with body condition score (BCS). Multiparous cows with a higher BCS (≥3.75) had a greater risk of developing milk fever and multiple diseases than cows with lower BCS, and the risk was reduced in cows supplemented with negative DCAD diets (–121 to –100 mEq/kg; Serrenho et al., 2021). Although the effects of adiposity and energy metabolism on Ca dynamics, and vice versa, have not been deeply investigated, excessive BCS has been previously associated with clinical hypocalcemia (e.g., 4.3 times more incidence of milk fever in fat cows; Heuer et al., 1999). A recent study demonstrated that the metabolic acidosis caused by decreasing DCAD from –70 to –180 mEq/kg reduced the release of insulin after an intravenous glucose tolerance test was applied and tended to increase the release of plasma nonesterified fatty acids (NEFA) after an insulin challenge was performed in cows (Vieira-Neto et al., 2021). Moreover, abundance of proteins involved in the regulation of protein synthesis and gluconeogenesis in the liver was reduced, and abundance of proteins that regulate lipolysis in the adipose tissue was increased (Vieira-Neto et al., 2021). Although this needs to be further investigated, highly acidogenic diets in the prepartum period might negatively impact energy metabolism in dairy cows, and this aligns with a meta-analysis by Santos et al. (2019) indicating that a DCAD below –150 mEq/kg might not be necessary for multiparous cows.

### Energy in the prepartum period diet

Because dairy cows decrease DMI around parturition, increasing the energy density of the prepartum diet would be considered a logical approach to ameliorate the negative energy balance and improve lactational performance of the cows (Figure 2; Grummer, 1995). However, increasing energy intake above NRC (2001) requirements for the prepartum period might be deleterious for performance and health after parturition. Dann et al., (2006) demonstrated that controlling energy intake (80% and 100% vs. 150% of NRC 2001 requirements) resulted in decreased plasma NEFA and β-hydroxybutyrate (BHB) concentrations, and increased DMI of cows in the first 10 DIM. Recently, Richards et al. (2020) demonstrated that switching cows from a lower energy (1.34 Mcal/kg of NE_L_) to a higher energy diet (1.61 Mcal/kg of NE_L_) in the dry period (i.e., transitioning from the far-off to close-up group) had little effect on lactational performance compared with a lower energy diet fed over the entire dry period. Furthermore, higher energy diets did not benefit milk production but increased blood NEFA and BHB concentrations (Richards et al., 2020), which indicates a poor adaptation to the lower energy balance and a higher body tissue mobilization after parturition.

**Figure 3.**
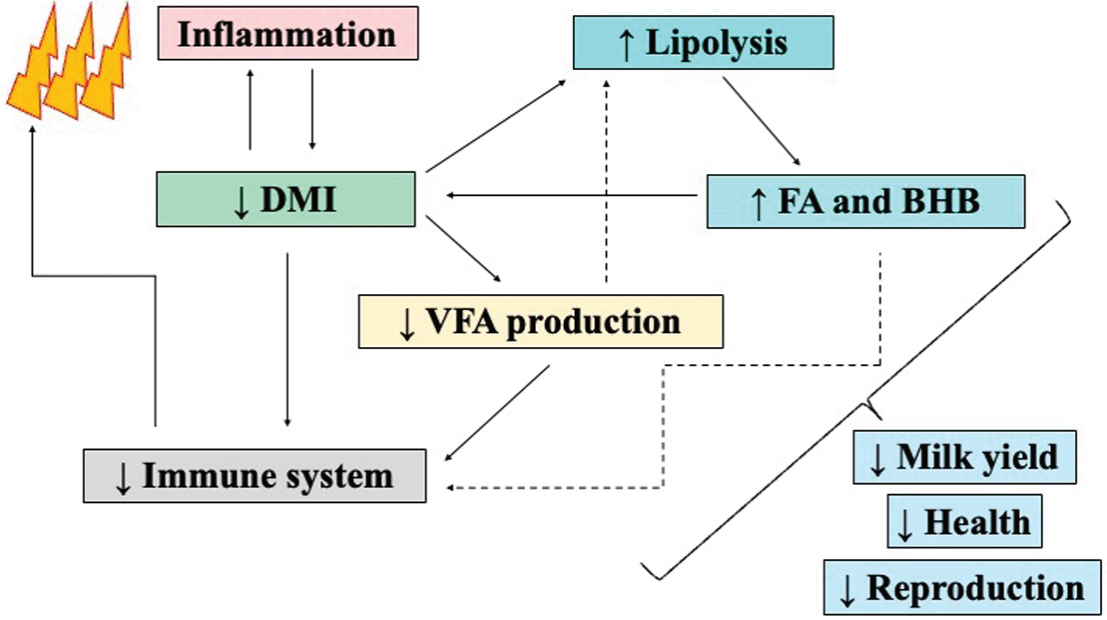
Diagram of the integration of immune activation, dry matter intake, and lipolysis occurring peripartum. The depletion of activity of the immune system will ultimately affect milk yield, health, and reproduction of dairy cows. DMI = dry matter intake; FA = plasma total fatty acids; BHB = plasma β-hydroxybutyrate.

Studies have indicated that increasing NEFA blood concentration inhibits immune function in dairy cows (Figure 3). Number of cells, chemotactic ability, phagocytic activity, and oxidative burst activity of polymorphonuclear cells are reduced by increased NEFA (Contreras et al., 2018). In addition, NEFA can increase reactive oxygen species and apoptosis of neutrophils, decrease proliferation and stimulation of peripheral blood mononuclear cells, decrease IgM and IFN-γ secretion, and reduce the response of lymphocytes (Contreras et al., 2018). Feeding dairy cows with 33.7 Mcal/d of ME (i.e., higher energy) compared with 23.6 Mcal/d of ME (i.e., lower energy) impaired gluconeogenesis and FA oxidation by downregulating mRNA expression of key enzymes involved in glucose synthesis and beta-oxidation in the liver (Selim et al., 2014). Thus, decreased blood glucose concentration and oxidation of NEFA in the liver would negatively influence cows to respond to inflammatory stimuli during the onset of lactation. Overfeeding cows during the dry period resulted in a greater degree of lipogenesis prepartum (i.e., increased expression of classic lipogenic gene in subcutaneous adipose tissue), and increased blood concentration of BHB postpartum, but no differences in the quantity of insulin receptors (i.e., protein abundance) and inflammatory markers were observed (Mann et al., 2016). Feeding controlled energy diets (below 1.45 Mcal/kg NE_L_) to prepartum dairy cows might be the best choice to reduce blood concentrations of NEFA and BHB. Additionally, lactational performance (i.e., DMI and milk production) and overall animal health could improve.

### Amino acids in the prepartum diet

Recommendations for protein nutrition in the prepartum period are not well established because there is a lack of conclusive data to define requirements for transition dairy cows. Increasing the amount of dietary rumen undegradable protein (RUP) from 29% to 39% (as a % of crude protein; CP) prepartum failed to improve DMI and milk production in the subsequent lactation of dairy cows (Hartwell et al., 2000). Conversely, increasing dietary RUP during prepartum from 15% to 37% (as a % of CP) resulted in greater milk production, milk protein and casein yields, and fertility of dairy cows postpartum (Rodney et al., 2016). Variability in responses from increased RUP in diets may be related to the source and quality of feed ingredients, and therefore, the potential of RUP to enhance or not the amino acid (AA) profile and supply of metabolizable protein (MP). In a meta-analysis, Husnain and Santos (2019) demonstrated that primiparous cows may require greater amounts of MP than multiparous cows during the prepartum period. Diets with 14% to 15% CP are enough to supply 1,100 g/d of MP, which seems to be a good target in the diet of primiparous cows during the prepartum period. On the other hand, the performance of high-producing multiparous cows was not improved with diets over 800 g/d of MP fed in the prepartum. It is important to note that performance of dairy cows might be dependent on an interaction between dietary MP concentration pre- and postpartum. For instance, cows fed a lower MP diet prepartum (80 g/kg of DMI; 12% CP) followed by a lower MP diet postpartum (102 g/kg of DMI; 16% CP) decreased DMI, milk yield, and milk component yields after parturition compared with cows fed a higher MP diet postpartum (119 g/kg of DMI; 19% CP) and with cows fed a higher MP diet prepartum (101 g/kg of DMI; 15% CP), regardless of MP postpartum (Farahani et al., 2019). These results indicate that increasing dietary protein postpartum might improve performance of cows fed lower protein diets prepartum.

Studies also tried to identify the effects of formulating diets for ruminants focusing on the balance of limiting AA. Researchers observed a 2.0 kg/d of DMI increase in cows fed a 15.6% CP diet prepartum compared with a 13.8% CP diet, and a tendency for increased DMI prepartum (1.6 kg/d) by methionine (Met) supplementation of the higher CP diet (Cardoso et al., 2021). Additionally, cows on the higher CP diets had greater IL-1 concentration in blood postpartum and tended to produce 1.75 kg/d more milk than cows fed the low protein diet during the first 45 d of lactation, regardless of Met supplementation. Inflammation and oxidative stress mitigation by Met supplementation in prepartum diets have also been reported by others. For instance, plasma concentrations of IL-6, neutrophil phagocytosis activity, and oxidative burst were enhanced by Met supplementation to achieve a lysine to Met ratio of 2.8:1 during prepartum (Batistel et al., 2017). Lysine (Lys) has also been supplemented in prepartum diets, but the results are not consistent. Fehlberg et al. (2020) demonstrated increased energy-corrected milk and milk components yields with dietary prepartum supplementation of Lys (1.0 g of digestible Lys per kg of DMI). Lee et al. (2019), on the other hand, did not observe improvements in lactational performance and health of cows fed prepartum with similar amounts of digestible Lys. Variability in these responses is somewhat expected since the benefits of Lys supplementation are more evident in Lys deficient diets (e.g., diets with corn-based diets with reduced soybean-meal; Lobos et al., 2021). In summary, these results suggest increased performance and immune function in periparturient cows fed higher protein diets, regardless of amino acid supplementation, with potential benefits in performance and health during the lactation.

### Phytonutrients

Feeding phytonutrients (PN) can be an alternative to bolster immune function and antioxidant activity in livestock. Certain PN interact with transient receptor potential (TRP) channels which are a group of ion channels expressed within immune cells, intestine, and other tissues (Holzer, 2011). Binding of these channels with PN can produce various physiological outcomes, both pro- and anti-inflammatory. For example, eugenol, commonly found in the essential oil (EO) of clove, has been documented to bind to ion channels (i.e., TRPV1 and TRPV3) leading to anti-inflammatory and antioxidant properties; while cinnamaldehyde (found in cinnamon) mediates with another channel (i.e., TRPA1) and serves as an immune enhancer (Vriens et al., 2008). Capsaicin is another compound that can bind with TRPV1 and modulates immune response and improves mucosal blood flow; potentially modifying lymphocytes, macrophages, and neutrophils by mediating cytokine and antibody levels (Oh et al., 2017a). In monogastric species, increased digestive secretions and nutrient absorption, antioxidant activity and reduced pathogen stress within the gut while feeding EO have been observed (Zeng et al., 2015). A comprehensive review of studied EO and their effect on ruminant immunity are beyond the scope of this paper, but have been documented elsewhere (Oh et al., 2017b).

Capsaicin, tannins, curcumin, garlic, grape, and juniper extracts rank among the most well-researched PN in cattle. Effects range from increases in CD4+ cells, neutrophils, eosinophils, total plasma antioxidants, and superoxide dismutase and glutathione peroxidase activity, to decreased concentrations of haptoglobin, cortisol, and thiobarbituric acid reactive substances (Oh et al. 2013, 2015, 2017a). Hemp and hemp oils containing cannabinoids, terpenes, and flavonoids are known to interact with the endocannabinoid system (ECS) through cannabinoid receptors found on mammalian cells and tissues (e.g., brain, heart, blood vessels, liver, lungs, and digestive system; Mackie, 2008), including immune cells (e.g., T cells; Hartsel et al., 2019). The ECS regulates the inflammation response and may potentially be influenced by feeding hemp or hemp extracts. However, in vivo research, particularly in cattle, is required to substantiate these claims (Hartsel et al., 2019).

**Figure 4.**
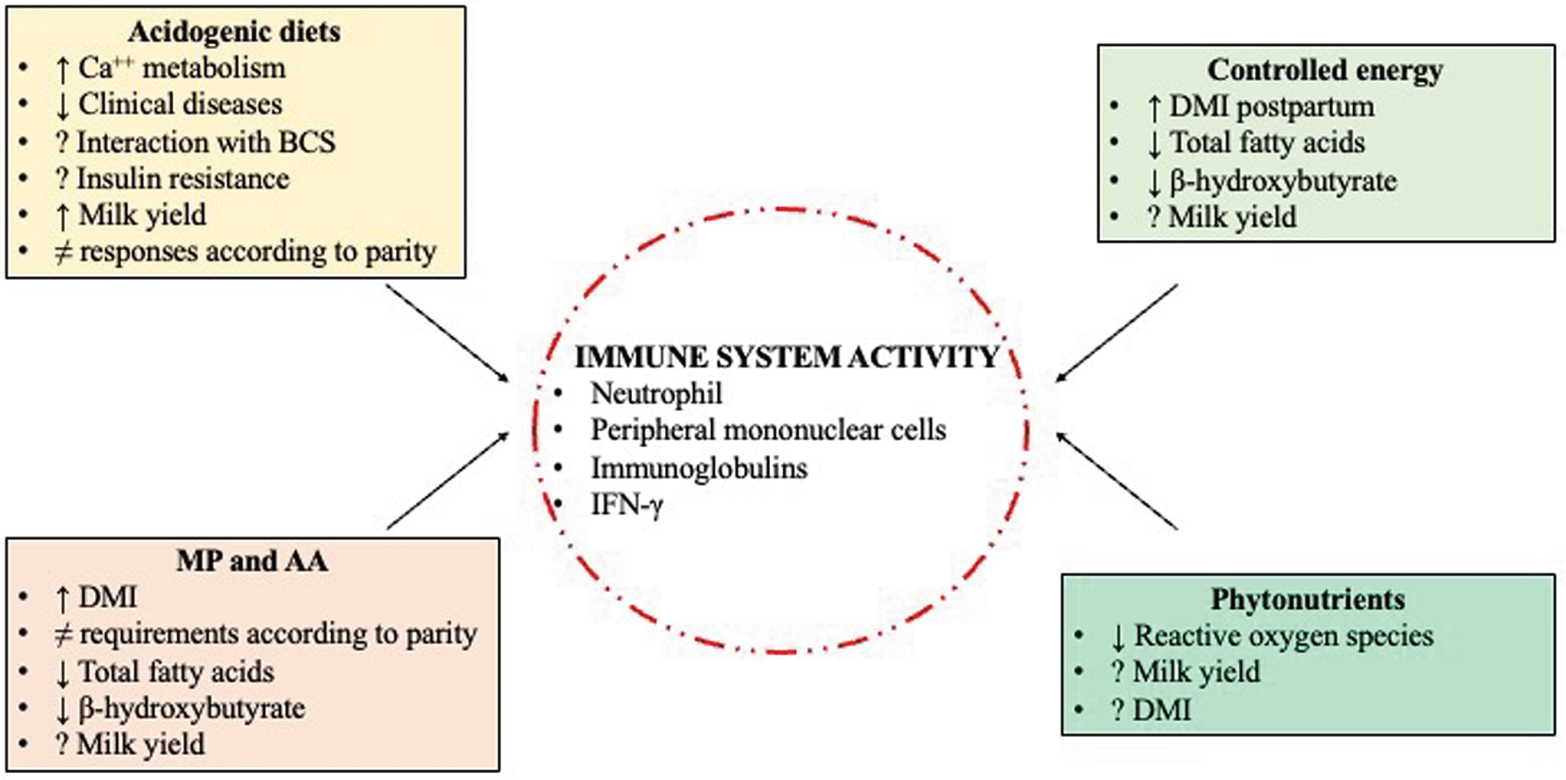
Diagram summarizing the main effects of nutritional strategies with a potential to regulate the activity of the immune system in dairy cows. Production and metabolic responses to acidogenic and controlled energy diets, metabolizable protein (MP), amino acids (AA), and phytonutrients supplementations are also described. Ca^++^ = calcium; BCS = body condition score; DMI = dry matter intake.

Macroalgae and their bioactive ingredients offer another vast area of potential PN. Within the realm of ruminant livestock, a brown algal species, *Ascophyllum nodosum*, has been investigated for its high content of phylorotannins and increases in superoxide dismutase activity (Fike et al., 2001). Numerous other compounds found within brown, green, and red algae have demonstrated potent antimicrobial activity and may help lower endotoxin load in the gut through the inhibition of antagonistic intestinal microbial populations (Pérez et al., 2016). Polyphenolic compounds extracted from *Porphyra denata* reportedly inhibit the inflammatory mediators nitric oxide, inducible nitric oxide synthase, and nuclear factor-κβ (Kazłowska et al., 2010). Sulfonated glycans found in brown algal fucoidans facilitate leukocyte migration and activation, as well as cytokine regulation; and have been researched in pursuit of developing new carbohydrate-based drug therapies to modulate acute and chronic inflammatory responses in humans (Pomin, 2015). These PN may be useful tools to manipulate the dairy cow’s immune system in times of suppression or detrimental activation. Speculatively and in agreement with the recommendations of Bradford et al. (2015), feeding an immune boosting PN to immunocompromised close-up cows followed by a PN that increases antioxidant activity post calving could be beneficial during the transition period. However, far more research is required to understand the timing and extent to which nutrition can be used to regulate the bovine immune system (Figure 4).

### Future perspectives

Future research should investigate how energy, protein, and mineral metabolisms are integrated with transition cow nutrition, and how that interacts with the immune system. Ideally, hormonal, immunological, and metabolic parameters of periparturient cows should be monitored hourly to detect changes and trends in a shorter timeframe with a larger number of samples. Additionally, the investigation of the effects of phytonutrients and other additives on the immunometabolism of dairy cows should be performed within a broader range of diets, and the exact mechanisms of interaction between nutraceutical compounds, diet, and host must be determined. Regarding the use of acidogenic diets in the prepartum period, future research should investigate the effects of DCAD levels on insulin resistance and glucose metabolism, considering possible associations with the somatotropic axis regulation (e.g., IGF-1 and growth hormone concentrations) and interactions with dietary energy. Lastly, only a few studies were adequately designed to evaluate the interaction between energy and protein in prepartum diets. Taken together, all these approaches may allow us to better understand the regulation of immunometabolism in dairy cows.

## Conclusions

Considering the importance of dietary nutrients to maintain homeostasis and modulate the immune system, dry matter intake seems to be the most important factor determining nutrition and health of dairy cows. Thus, the adoption of feeding strategies herein described would positively contribute to lactational performance by increased intake in the pre- or postpartum periods. Additionally, the use of nutraceuticals such as phytonutrients may be beneficial to intestinal health and immune regulation in dairy cows.

## About the Authors

**Leoni F. Martins** is a PhD student in the Department of Animal Science at The Pennsylvania State University under the mentorship of Dr. Alexander N. Hristov. He has a master’s in Veterinary Sciences from the Federal University of Uberlandia, Brazil and has worked as a research scholar at Purdue University, IN. His main research interests are in precision feeding and the application of feeding strategies to improve performance and health of dairy cows while reducing the waste of nutrients.

**Derek E. Wasson** is a PhD student in Dr. Alexander Hristov’s dairy nutrition laboratory. His research is focused on feeding seaweed to cattle, primarily for enteric methane inhibition, but also for improved immune function. He also has a practical background, growing up on a family dairy farm in PA and four years of industry experience working for animal nutrition companies in the Northeast and Midwest United States.

**Alexander N. Hristov** is a Distinguished Professor of Dairy Nutrition in the Department of Animal Science at The Pennsylvania State University. He has a PhD in Animal Nutrition from the Bulgarian Academy of Agricultural Sciences and has worked as a research scientist in his native Bulgaria, USDA-ARS Dairy Forage Research Center in Madison, WI, and the Ag Canada Research Center in Lethbridge, AB. He was on the faculty at the Department of Animal and Veterinary Science, University of Idaho, from 1999 to 2008 and is at Penn State since 2008. His main research interests are in the areas of protein/amino acid nutrition of dairy cattle and mitigation of nutrient losses and gaseous emissions from dairy operations. Corresponding author: anh13@psu.edu
